# Metagenomic Study of the Grapevine Decline Detected a Cocktail of Fungi Associated with Grapevine Trunk Diseases

**DOI:** 10.3390/plants14243722

**Published:** 2025-12-05

**Authors:** Daria Karpova, Daria Belkina, Elena Porotikova, Evgeniya Yurchenko, Svetlana Vinogradova

**Affiliations:** 1 Skryabin Institute of Bioengineering, Research Center of Biotechnology of the Russian Academy of Sciences, Leninsky Prospect, 33, Build. 2, 119071 Moscow, Russia; 2 North Caucasian Federal Scientific Center of Horticulture, Viticulture, Wine-Making, 40 Years of Victory Street, Build. 39, 350901 Krasnodar, Russia

**Keywords:** *Vitis vinifera*, grapevine decline, grapevine trunk diseases, microbiome, amplicon sequencing, Phomopsis dieback, Botryosphaeria dieback, Esca complex

## Abstract

This study analyzed the microbiome of three varieties differing in genotype and technical purpose: Cristal, Riesling, and Avgustin, all exhibiting decline symptoms of unknown etiology. A total of 92 symptomatic and asymptomatic grapevines were analyzed using ITS and 16S rRNA amplicon sequencing and molecular genetic methods. Phytoplasmas and the pathogenic bacteria *Xylella fastidiosa* and *Xylophilus ampelinus* were not present in the samples. The decline symptoms were associated with a cocktail of fungal pathogens that cause grapevine trunk diseases. In particular, the analysis revealed the causative agents of Botryosphaeria dieback (*Sphaeropsis* spp. and *Botryosphaeria* spp.), fungi associated with the Esca complex (*Phaeomoniella* spp., *Phaeoacremonium* spp., *Inonotus* spp., *Seimatosporium* spp., *Stereum* spp., and *Cadophora* spp.), and the causative agents of Phomopsis dieback (*Diaporthe* spp.). The symptoms of decline may be increased by several facultative grapevine pathogens that have been identified in microbiome (genera *Stemphylium*, *Alternaria*, *Aspergillus*, *Penicillium*, *Talaromyces*, and *Fusarium*). The metagenomic data of the grapevine microbiome provides opportunities for developing disease control strategies, which is important for the sustainable management of vineyards.

## 1. Introduction

Grape (*Vitis vinifera* L.) is one of the most important agricultural crops, with a centuries-old history of cultivation and a wide geographic range. In 2024, Russia ranked 17th in the world in vineyard area (107,000 hectares) [1]. Krasnodar region is Russia’s largest viticultural territory, occupying most of the flatland territory of the Western Ciscaucasia. Viticulture is one of the priority sectors of agricultural crop production in Krasnodar region [2].

With climate change, viticulture is facing increased aggressiveness of known pathogens and the expansion of new ones, including those that can cause economically damaging systemic infections [3,4,5,6]. One of the symptoms of a systemic infection in grapevine is decline. Affected plants exhibit chlorosis and necrosis on leaves, stunted growth, wood necrosis and decay, death of spurs, arms, and cordons. Symptoms develop slowly and may not manifest for a long time [7]. Depending on the grapevine variety, genotype, vineyard management system, the influence of abiotic factors, and infection by other pathogens, the manifestation of symptoms on affected plants may vary and typical symptoms may be masked. For example, under the combined influence of pathogens and adverse environmental conditions, shoots may dry out up to the complete death of the vine [8]. Additionally, the order in which pathogens infect the host during coinfection, as well as their trophic specialization, can affect the nature and severity of the developing disease [9]. Decline indicates profound disturbances in plant physiology. The primary cause of decline is damage to the vascular system, resulting in disruption of water transport, which leads to weakening of the plant [10].

Selecting an effective disease control strategy requires accurate diagnostics of the pathogen. The difficulty in determining the etiology lies in the wide range of pathogens that can contribute to the development of disease. The causative agents of decline can also be fungi [11], bacteria [12], viruses [13], and phytoplasmas [14]. Dangerous systemic infections leading to decline include grapevine trunk diseases (GTDs) which are caused by a complex of fungal pathogens and found in all wine-growing regions of the world. GTDs include several diseases which differ in symptoms and species of pathogenic fungi. These diseases cause plant dieback by damaging vascular tissues [15]. Among the causative agents are species of the Botryosphaeriaceae family (dieback and canker) [16], *Eutypa lata* (Pers.) Tul. & C. Tul. (Eutypa dieback) [17], *Cylindrocarpon* spp. and *Campylocarpon* spp. (Black foot) [18], *Diaporthe ampelina* (Berk. & M.A. Curtis) R.R. Gomes, Glienke & Crous (Phomopsis dieback or excoriosis) [19], and a number of pathogenic fungi responsible for the development of Esca disease [18]. *E. leptoplaca* (Durieu & Mont.) Rappaz, *Cryptovalsa ampelina* (Nitschke) Fuckel, *Cryptosphaeria pullmanensis* Glawe, and some other species of the genera *Diatrype*, *Diatrypella*, and *Eutypa* have also been shown to be involved in the development of GTDs [20]. The etiology of Esca disease is particularly intricate. It is divided into vascular disease and wood rot. Vascular disease can manifest itself as three vascular syndromes: brown wood streaking, Petri disease, and grapevine leaf-stripe disease [18,21]. As a rule, the causative agents of these three syndromes are *Phaeomoniella chlamydospora* (W. Gams, Crous, M.J. Wingf. & Mugnai) Crous & W. Gams and *Phaeoacremonium aleophilum* W. Gams, Crous, M.J. Wingf. & Mugnai, while the causative agents of wood rot are *Fomitiporia mediterranea* M. Fisch. and other species of the genera *Fomitiporia*, *Fomitiporella*, *Inocutis*, etc. [21,22].

Currently, the most powerful tools for analyzing microbial communities and determining the etiology of systemic diseases are represented by molecular methods, including, in particular, high-throughput sequencing technologies [23]. Metagenomic technologies are actively used in phytosanitary research, as they make it possible to identify uncultivated or difficult-to-cultivate microorganisms [24,25,26].

Because decline is a fairly common symptom and its etiology remains unclear, the aim of this study was to conduct a comprehensive microbiome analysis of affected grapevines using high-throughput sequencing and molecular genetic diagnostics.

## 2. Results

### 2.1. Decline Symptoms of Grapevines

**Figure 1:**
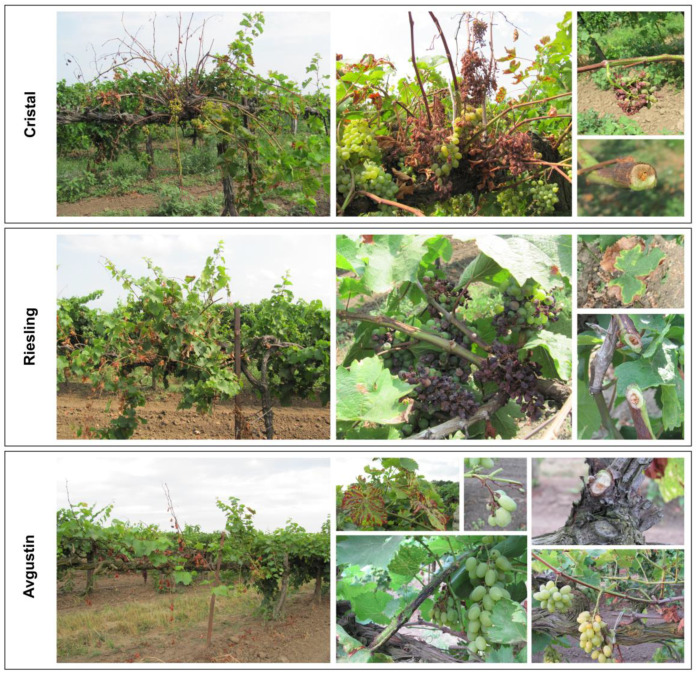
Decline symptoms in three grapevine varieties in August 2023: Cristal (Svetly Put Lenina), Riesling (Svetly Put Lenina), and Avgustin (Kurchanskaya).

A total of 92 grapevine samples were collected from the beginning of BBCH 69 phase until harvest (Figure S1). Among all the grapevines collected, the most common symptoms were necrosis, complete drying out of leaves, and drying out of part of the shoot (usually the apex) or the entire shoot (Figure 1, Table S1). Minor lesions, manifested as necrosis and bark cracking which merged over time, were also observed on the shoots. Symptoms on the leaves manifested as necroses beginning at the leaf margin and increasing in area over time, as well as discoloration of the interveinal spaces and chloroses which subsequently necrotized, while the veins remained green. Furthermore, a characteristic feature was uneven lignification of the shoots, accompanied by necroses of the vascular system which were clearly visible in the cross-sections of the shoots, especially at the first internodes. The Avgustin grapevines exhibited partial rachis necrosis. The clusters themselves on affected plants of all varieties became wilted, shriveled and partially or completely dried out. Symptoms were often localized to closely positioned vines or to a single branch of the vine. By harvest time, bunches of bare, dried-out vines with completely dried or fallen leaves could be observed on affected plants.

During examinations of the same affected bushes of the Riesling and Cristal cultivars the following year (2024), no decline symptoms were observed on the grapevines remaining after pruning the dried vines when performing agrotechnical measures.

### 2.2. Microbiome of Grapevines with Decline Symptoms

Sequencing of the prepared 92 16S rRNA libraries and 92 ITS libraries was successfully fulfilled on an Illumina NovaSeq 6000 platform. In total, 9,545,875 bacterial paired reads (average 103,760 per sample) and 6,118,146 fungal paired reads (average 66,502 per sample) were obtained (Table S2). After denoising, 3,272,453 ASVs for 16S rRNA and 5,384,375 ASVs for ITS were obtained. All ASVs were taxonomically annotated and filtered, which made it possible to identify 37 bacterial genera (Table S3) and 147 fungal genera (Table S4).

#### 2.2.1. Taxonomic Composition of Fungal Communities

**Figure 2:**
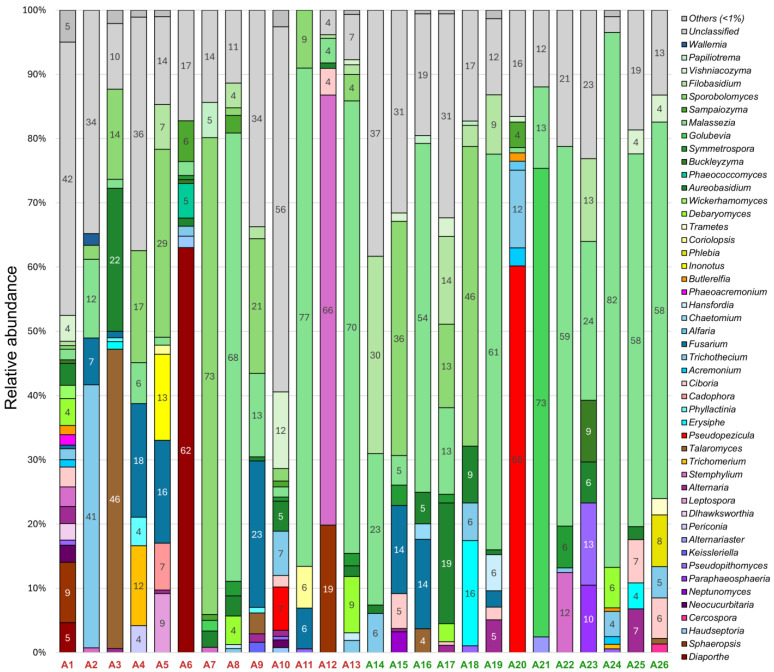
Fungal community composition in Avgustin grapevines at genus level with relative abundance > 1%. Samples with (A1–A13) and without (A14–A26) decline symptoms are marked in red and green, respectively.

In 26 grapevines of the Avgustin variety, 48 fungal genera with a relative abundance of over 1% were detected (Figure 2). Two-thirds of them (33 genera) belonged to the Ascomycota division, the rest (15 genera) belonged to the Basidiomycota division. Yeasts and yeast-like fungi were predominant: *Malassezia* spp. (1–82% in 24 samples), *Sporobolomyces* spp. (1–73% in 14 samples), *Filobasidium* spp. (1–30% in 11 samples), and *Aureobasidium* spp. (1–22% in 16 samples). In addition, a large amount of filamentous fungi such as *Trichothecium* spp. (1–41% in 12 samples) and *Fusarium* spp. (1–23% in 10 samples) was present.

Of the plant pathogens, fungi of the genera *Pseudopezicula*, *Erysiphe*, *Fusarium*, *Phyllactinia*, *Phaeoacremonium*, *Cadophora*, *Pseudopithomyces*, *Butlerelfia*, *Sphaeropsis*, and *Diaporthe* were detected in the samples. Notably, *Pseudopezicula* spp., *Erysiphe* spp., *Pseudopithomyces* spp., and *Butlerelfia* spp. were found in grapevines regardless of the presence of decline symptoms. On the contrary, *Fusarium* spp. predominated in symptomatic vines (1–23% in seven samples) and was detected only in three visually healthy samples (3–14%). The following pathogens were identified only in samples with decline symptoms: *Diaporthe* spp.—in two samples with a relative abundance of 5% and 62%, *Sphaeropsis* spp.—in two samples (9–19%), *Phyllactinia* spp.—in three samples (1–4%), *Cadophora* spp.—in one sample (7%), and *Phaeoacremonium* spp.—in one sample (2%).

**Figure 3:**
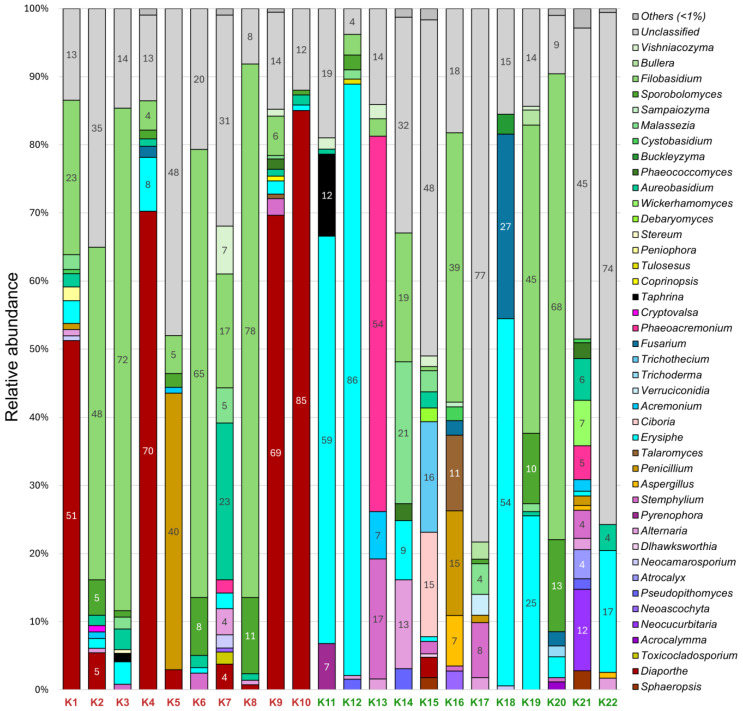
Fungal community composition in Cristal grapevines at genus level with relative abundance > 1%. Samples with (K1–K10) and without (K11–K22) decline symptoms are marked in red and green, respectively.

In 22 grapevines of the Cristal variety, 42 fungal genera with a relative abundance of more than 1% were identified (Figure 3). As in the previous variety, most of them (30 genera) belonged to the Ascomycota division, and the remaining 12 genera belonged to the Basidiomycota division. The most represented genera were yeast-like *Filobasidium* spp. (1–78% in 16 samples) and *Aureobasidium* spp. (1–23% in 14 samples). In addition, among the dominant taxa were phytopathogenic *Erysiphe* spp. (1–86% in 17 samples) and *Diaporthe* spp. (1–85% in 9 samples).

It is interesting that the *Erysiphe* genus was more represented in grapevines without decline symptoms (1–86%) than in those with symptoms (1–8%). In only five samples, this genus was either completely absent or its relative abundance was less than 1%. In contrast, the *Diaporthe* genus dominated in grapevines with decline symptoms; it was found in eight out of ten such samples with a relative abundance of up to 85%. Only one sample without decline symptoms was found to contain *Diaporthe* spp. with an abundance of more than 1%. Two samples were infected with an Esca-associated fungi *Phaeoacremonium* spp. Note that its relative abundance in grapevine without decline symptoms was 54%, while in grapevine with symptoms it was 2%. *Fusarium* spp. was more common in vines without decline symptoms (2–27% in three samples) compared to symptomatic vines (2% in one sample). In several samples, the abundance of plant pathogens *Sphaeropsis* spp., *Pseudopithomyces* spp., and *Cryptovalsa* spp. was less than 3%.

**Figure 4:**
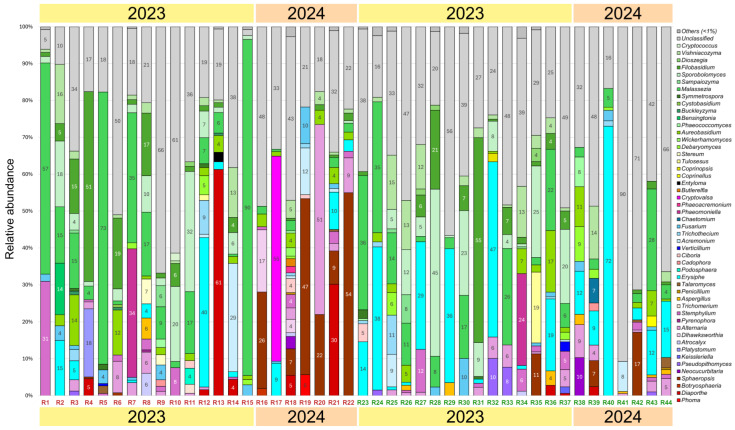
Fungal community composition in Riesling grapevines at genus level with relative abundance > 1%. Samples with (R1–R22) and without (R23–R44) decline symptoms are marked in red and green, respectively.

A total of 44 grapevines of the Riesling variety were found to be infected with 50 fungal genera with a relative abundance of more than 1%: 33 from the Ascomycota division and 17 from the Basidiomycota division (Figure 4). The dominant genera in the samples were yeasts and yeast-like fungi *Malassezia* spp. (1–90% in 38 samples), *Filobasidium* spp. (1–55% in 31 samples), *Sporobolomyces* spp. (1–45% in 27 samples), *Aureobasidium* spp. (1–17% in 28 samples), and *Vishniacozyma* spp. (1–16% in 28 samples). In addition, a high abundance of fungi from the genera *Alternaria* (1–51% in 26 samples) and *Stemphylium* (1–31% in 21 samples) was detected. Another dominant group consisted of phytopathogenic fungi *Erysiphe* spp. (1–72% in 26 samples) and *Sphaeropsis* spp. (1–54% in 11 samples).

As in the Cristal grapevines, the presence of *Erysiphe* spp. was not associated with the manifestation of decline symptoms. On the contrary, *Sphaeropsis* spp. predominated in symptomatic samples: in eight of them, its relative abundance was 1–54%, whereas in three asymptomatic samples it was less than 17%. Another pathogenic fungus, *Diaporthe* spp., was also more abundant in grapevines with decline symptoms (2–61% in six samples) compared to visually healthy grapevines (1–3% in three samples). *Fusarium* spp. were found in 17 samples at the abundance of 1–10%, regardless of symptom manifestation. Some plant pathogens were detected at the abundance of more than 1% exclusively in grapevines with decline symptoms, but no more than in one plant. These were fungi of the genera *Cryptovalsa* (55%), *Phoma* (6%), *Podosphaera* (4%), *Entyloma* (3%), *Botryosphaeria* (2%), *Butlerelfia* (2%), and *Phaeoacremonium* (2%). Other pathogenic genera were found in grapevines with and without decline symptoms. They included *Phaeomoniella*, *Verticillium, Pseudopithomyces*, and *Cadophora*.

#### 2.2.2. Functional Properties of the Fungal Communities

**Figure 5:**
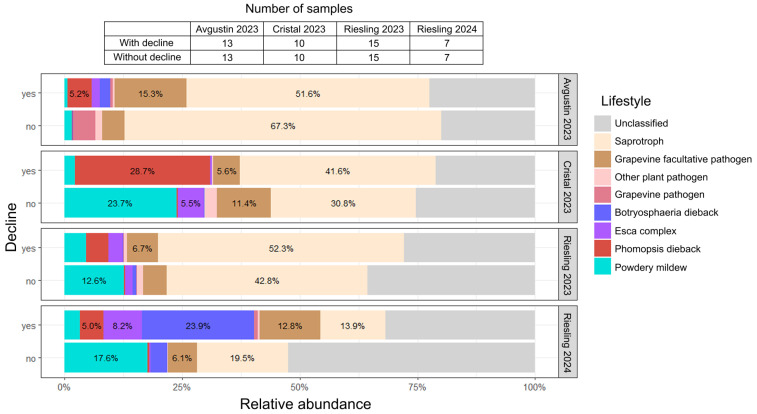
Functional properties of the fungal communities in grapevine with and without decline symptoms. The diagram is based on the relative abundance of each fungal genus in each sample. FungalTraits database and information on involvement in grapevine diseases were used to assign fungi to groups according to their lifestyle (Table S5).

Analysis of symptomatic and asymptomatic plants from three varieties allowed us to assign the identified fungal genera to 12 lifestyle groups according to FungalTraits database (Figure S2). Further analysis of fungal genus representation was conducted based on their involvement in grapevine diseases (Table S5). A significant proportion of the fungal communities of grapevines with decline symptoms in the studied varieties was represented by the causative agents of two diseases, Phomopsis dieback and Botryosphaeria dieback, represented primarily by *Diaporthe* spp. and *Sphaeropsis* spp., respectively (Figure 5). In all cultivars, the Phomopsis dieback causative agent was found only in grapevines with decline symptoms, where its abundance reached 28.7%. Another dominant pathogen, the causative agent of Botryosphaeria dieback, was also more represented in symptomatic vines (up to 23.9%) compared to visually healthy ones.

The Avgustin grapevines with decline symptoms were infected with pathogens of various GTDs (Botryosphaeria dieback, Esca complex, and Phomopsis dieback); in total, their abundance was about 9%. In visually healthy vines of the same cultivar, these pathogen groups were almost absent (<1%). Furthermore, symptomatic Avgustin samples had a higher representation of facultative grapevine pathogens (15.3%) compared to samples without decline symptoms (4.8%).

In grapevines of the Cristal and Riesling varieties, the causative agent of powdery mildew was prevalent, and its abundance was higher in vines without decline symptoms (12.6–23.7%) than in symptomatic vines (2.2–4.5%). In grapevines with decline symptoms of the 2023 Cristal variety, the most common pathogen was *Diaporthe* spp., the causative agent of Phomopsis dieback (28.7%). In asymptomatic vines, the abundance of this pathogen was less than 1%; however, they were characterized by a higher abundance of the causative agent of powdery mildew (23.7%) and Esca complex fungi (5.5%).

In the 2023 Riesling grapevines, a higher abundance of pathogens from the GTDs complex was observed in samples with decline symptoms (8%) compared to visually healthy vines (2.7%). The predominant pathogens in the symptomatic 2024 Riesling grapevines were the GTDs complex fungi, in particular, the causative agents of Botryosphaeria dieback (23.9%). The abundance of pathogens from the Esca complex and Phomopsis dieback was 8.2% and 5.0%, respectively. In visually healthy vines of this group, the total abundance of GTDs pathogens was approximately 4%.

#### 2.2.3. Taxonomic Composition of Bacterial Communities

Bacterial genera identified by amplicon sequencing belonged to four phyla: Pseudomonadota, Actinomycetota, Bacillota, and Bacteroidota.

In 26 grapevines of the Avgustin variety, 22 bacterial genera with a relative abundance of more than 1% were identified (Figure S3). The dominant genera were *Staphylococcus* (3–48% in 22 samples), *Sphingomonas* (5–75% in 21 samples), and *Cutibacterium* (2–40% in 12 samples). In vines with decline symptoms, more prevalent were the genera *Pantoea* (2–60% in five symptomatic samples and 8% in one visually healthy sample) and *Pseudomonas* (2–21% in four symptomatic samples and 3% in one visually healthy sample).

In 22 grapevines of the Cristal variety, 20 bacterial genera with a relative abundance of more than 1% were detected (Figure S4). The predominant bacterial genera were *Staphylococcus* (12–63% in 19 samples) and *Sphingomonas* (6–53% in 19 samples). The genera *Pseudomonas* and *Streptomyces* were identified only in grapevines with decline symptoms. *Pseudomonas* spp. was detected in three samples with a relative abundance of 8–21%, and *Streptomyces* spp. was found in two samples (16–21%).

In 44 grapevines of the Riesling variety, 28 bacterial genera with a relative abundance of more than 1% were detected (Figure S5). The most prevalent bacterial genera were *Staphylococcus* (4–82% in 34 samples), *Sphingomonas* (2–67% in 23 samples), *Acinetobacter* (2–68% in 20 samples), *Cutibacterium* (3–53% in 18 samples), and *Rothia* (5–52% in 17 samples). Interestingly, in the 2024 Riesling grapevines, *Pseudomonas* spp. was found in five of seven samples with decline symptoms, with a relative abundance of 2–29%, and was not found in any of seven visually healthy plants.

None of the 92 samples were found to contain bacteria from the genera *Xylella* and *Xylophilus* which include the harmful grapevine pathogens *Xylella fastidiosa* Wells et al. and *Xylophilus ampelinus* (Panagopoulos) Willems et al.

#### 2.2.4. Comparative Community Analysis

**Figure 6:**
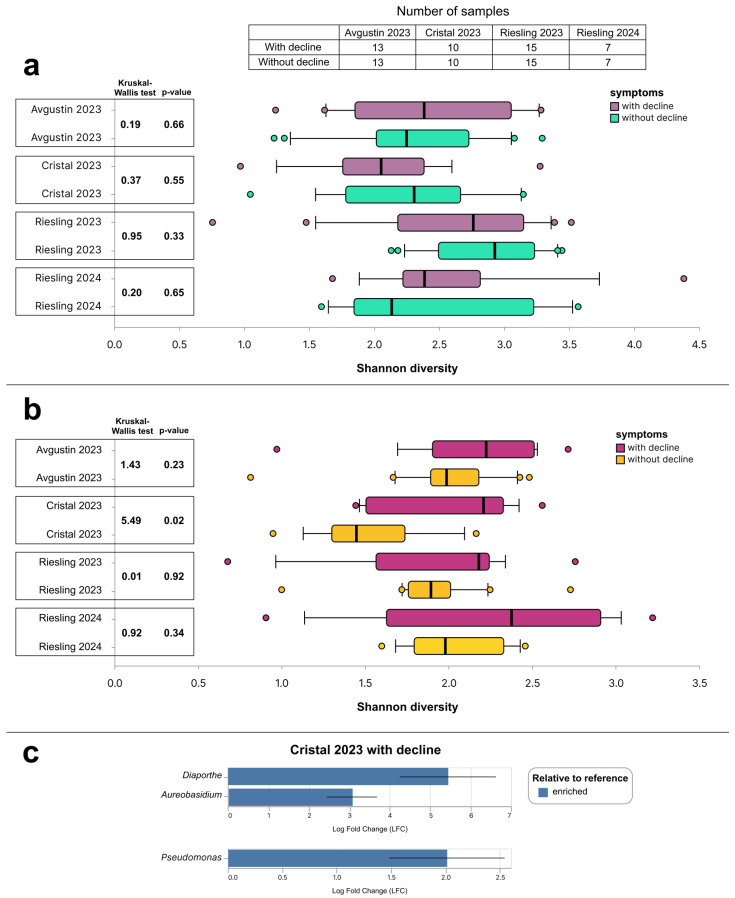
Alpha diversity of (**a**) fungal and (**b**) bacterial communities calculated using the Shannon index; (**c**) statistically significant results of the ANCOM-BC test (*p*-value < 0.05). The bold vertical line in the boxplot displays the median. The statistical differences between grapevines with and without decline symptoms were analyzed using the Kruskal–Wallis test.

The alpha diversity of bacterial and fungal communities in grapevine samples was studied using the Shannon index. The most diverse fungal communities were found in the 2023 Riesling grapevines (Figure 6a). The Kruskal–Wallis test did not show statistically significant differences in the alpha diversity of fungal communities of the studied varieties, with and without decline symptoms. The median value of the Shannon index of bacterial communities in all sample selections was higher in grapevines with decline symptoms than in those without them (Figure 6b). However, the results of the Kruskal–Wallis test showed statistically significant differences in the alpha diversity of samples with and without decline symptoms only for the Cristal cultivar of the 2023 harvest. Differential abundance analysis using ANCOM-BC at a *p*-value threshold of 0.05 showed that in the same group of Cristal 2023 samples, vines with decline symptoms were characterized by an increased abundance of the fungal genera *Diaporthe* and *Aureobasidium* and the bacterial genus *Pseudomonas* (Figure 6c). The ANCOM-BC results for other sample selections were not statistically significant.

Principal Coordinates Analysis (PCoA) based on the relative abundance of bacterial and fungal taxa revealed different levels of separation between grapevines with and without decline symptoms (Figure S6). PERMANOVA indicated significant differences in microbial communities for the Avgustin (*p*-value = 0.034, R^2^ = 6.1%) and Cristal (*p*-value = 0.006, R^2^ = 9.8%) grapevines, whereas Riesling showed no significant group separation (*p*-value > 0.077).

### 2.3. Screening for Phytoplasma Infection

Due to recent detection of 16SrV group phytoplasma in grapevines with decline symptoms in this region, all samples were analyzed for the presence of phytoplasmas. The results of real-time PCR with universal primers showed the absence of amplification, while the quantification cycles (Cq) of the internal control (chaperonin) ranged from 18 to 30 (Table S6). PCR with primers specific for the 16SrV group phytoplasma also showed the absence of phytoplasma infection in grapevine samples with and without decline symptoms.

## 3. Discussion

To determine the etiology of grapevine decline, we analyzed bacterial and fungal communities associated with grapevines with and without decline symptoms.

Analysis of 16S rRNA amplicon sequencing data showed a predominance of the genera *Sphingomonas* and *Staphylococcus* in all grapevine varieties. Other highly represented genera included *Curtobacterium*, *Pantoea*, *Pseudomonas*, *Acinetobacter*, and *Rothia*. These bacteria are typical representatives of the grapevine microbiome [27,28,29]. It is noteworthy that bacteria of the genus *Pseudomonas* are more abundant in grapevine samples with decline symptoms; statistically significant differences were found for the Cristal grapevines collected in 2023. Bacteria of the genus *Pseudomonas* include both pathogenic and nonpathogenic bacteria [30,31]. Therefore, to establish their role in the manifestation of decline symptoms and their association with other pathogenic microorganisms, it is necessary to isolate them in axenic culture and fulfill Koch’s postulates.

Among the bacterial genera identified are those that have the potential to serve as biological control agents. Thus, it was demonstrated that *Bacillus* spp. and *Pseudomonas* spp. had antagonistic activity against *Fomitiporia mediterranea* M. Fisch, one of the causative agents of GTDs [30,32]. *Enterobacter* spp. and *Pantoea agglomerans* strains reduced necrotic lesions when co-inoculated in planta with *Neofusicoccum parvum* (Pennycook & Samuels) Crous, Slippers & A.J.L. Phillips, which causes Botryosphaeria dieback [33]. Furthermore, it was shown that *Streptomyces* spp. strains reduced the level of infection by GTDs pathogens *Diplodia seriata* De Not., *P*. *chlamydospora*, and *Phaeoacremonium minimum* (Tul. & C. Tul.) Gramaje, L. Mostert & Crous [34].

It is worth noting that none of the 92 analyzed libraries were found to contain the pathogenic bacteria *Xylella fastidiosa* and *Xylophilus ampelinus* which are the causative agents of Pierce’s disease and bacterial blight of grapevine, respectively. Moreover, the 16SrV group phytoplasma which was previously found by us in this region on vines with decline symptoms was not detected in the samples. Thus, the etiology of decline symptoms is most likely to be associated with infection by a complex of fungi that are the causative agents of GTDs.

Analysis of ITS amplicon sequencing data revealed the presence of several fungal pathogens in the samples. The powdery mildew pathogen *Erysiphe* spp. was detected in 77% of the Cristal grapevines and 59% of the Riesling grapevines. Powdery mildew is one of the most prevalent diseases in vineyards in Krasnodar region [35].

One of the dominant pathogens in the analyzed grapevines was *Diaporthe* spp., which was almost never found in visually healthy plants. Representatives of this genus are the causative agents of Phomopsis dieback in grapevine [36]. Interestingly, typical symptoms of *Diaporthe* spp. infection were not detected on leaves, but shoot lesions (cankers, necroses, and bark cracking) were common. The absence of symptoms on the leaves may be associated with the fungicide treatment against the conidial stage.

Another widespread genus in samples with decline symptoms was *Sphaeropsis*, which is one of the potential causative agents of Botryosphaeria dieback. *Sphaeropsis* spp. and Esca complex fungi were detected in both symptomatic and asymptomatic plants. This suggests a latent progression of the disease, a possibility of which was demonstrated in previous studies [7]. Furthermore, inconsistent manifestation of symptoms from year to year is one of characteristic features of GTDs [37], which masks the true extent of the disease in the vineyard. 

It is known that abiotic stresses can facilitate the spread of GTDs pathogens and exacerbate symptoms manifestation [38,39,40]. Previously, GTDs symptoms in Russian vineyards were found to be associated with a number of genera, including *Diaporthe*, *Botryosphaeria*, *Stereum*, *Phaeomoniella*, and *Phaeoacremonium* that were identified in our study [41]. The spread of these pathogens may be associated with the increased frequency of extreme weather conditions in Krasnodar region amid global warming [42,43,44]. The harmfulness of GTDs can also be exacerbated by biotic stresses caused by infection with other pathogens. For example, a high abundance of *Erysiphe* spp., the causative agent of powdery mildew (up to 86%), was found in visually healthy Cristal and Riesling grapevines harvested from a single vineyard. This may indicate the presence of a significant reservoir of infection which is in a latent state. Grapevine infection with powdery mildew may be one of the factors in the development of GTDs in vines of the Cristal and Riesling varieties. Therefore, there is a high risk of developing decline symptoms on apparently healthy vines infected with *Erysiphe* spp.

Moreover, a number of saprotrophic fungal species, such as *Fusarium* spp. and *Alternaria* spp., can act as pathogens on weakened plants [3,4,45]. Therefore, the facultative grapevine pathogens identified in this study (the genera *Stemphylium*, *Alternaria*, *Aspergillus*, *Penicillium*, *Talaromyces*, and *Fusarium*) may contribute to the development of decline symptoms. However, members of the genera *Trichoderma*, *Chaetomium*, and *Fusarium* were used as biocontrol agents against *D*. *seriata* [46]. It was determined that *Fusarium lateritium* Nees in vitro degraded some toxins involved in the expression of GTD symptoms on leaves and was effective in protecting against *E*. *lata* [47,48]. In addition, yeast-like fungi *Debaryomyces* spp., *Vishniacozyma* spp., and *Yamadazyma* spp. were detected in the samples, which, according to literature data, may be antagonists of gray rot and anthracnose [49,50,51]. Information about potential biocontrol agents in the grapevine microbiome will form the foundation for future disease control strategies.

Thus, the data obtained from a comprehensive microbiome analysis of the Avgustin, Cristal, and Riesling varieties indicate the presence of a complex of the vascular system pathogens, including causative agents of GTDs. A mixed infection makes it difficult to assess the individual contribution of each pathogen. Future studies should include microbiological research to obtain axenic cultures of fungi associated with decline, followed by inoculation of healthy grapevines to fulfill Koch’s postulates. A promising approach that can be used in further research is the study of interactions within this complex of pathogenic microorganisms. This emphasizes the importance of whole-plant microbiome analysis for the development of reliable and effective protective measures that take into account the impact of biotic and abiotic factors.

## 4. Materials and Methods

### 4.1. Samples Collection

Samples were collected in Krasnodar region (Russia) from July to September in 2023–2024. Three grapevine cultivars, differing in genotype and technical purpose, were analyzed: Cristal, Riesling Weiss (Riesling), and Avgustin. The vineyards were 19–23 years old at the time of the sampling. Riesling and Cristal are wine grape cultivars widely grown in the vineyards of Krasnodar region. Riesling is a cultivar of the Western European eco-geographical group—convar. *occidentalis* subconvar. *gallica*, while Cristal is a Euro-Amur hybrid. Another variety, Avgustin, is a Euro-American hybrid and is a table grape cultivar with valuable consumer and selection qualities, such as increased resistance to major diseases and adverse abiotic factors.

Vine and leaf samples were collected from grapevines exhibiting decline symptoms and from asymptomatic grapevines. A total of 92 samples were collected. All samples were individually packaged and transported to the laboratory at +4 °C. Prior to DNA extraction, the samples were stored at −20 °C.

### 4.2. Library Preparation for 16S rRNA and ITS Amplicon Sequencing

For high-throughput sequencing, DNA was extracted from leaf and vine fragments using CTAB buffer [52]. DNA quality and quantity were monitored using a Nano-500 spectrophotometer (Allsheng, Hangzhou, China) and electrophoresis in a 1.2% agarose gel. Libraries for amplicon sequencing were prepared according to the 16S Meta-Genomic Sequencing Library Preparation protocol [53]. A fragment of the bacterial 16S rRNA gene (V4 variable region) was amplified with primers 515F (5′-GTGYCAGCMGCCGCGGTAA-3′) [54] and 806R (5′-GACTACHVGGGTATCTAATCC-3′) [53], and a region of the internal transcribed spacer (ITS) of fungi—with primers ITS1-f (5′-CTT GGT CAT TTA GAG GAA GTA A-3′) and ITS2 (5′-GCT GCG TTC TTC ATC GAT GC-3′) [55]. For PCR, we used the KAPA HiFi HotStart ReadyMix (2X) (Roche, Basel, Switzerland); for the ITS1-f/ITS2 primer pair, the number of amplification cycles was increased to 30. PCR products were purified using the Agencourt AMPure XP kit (Beckman Coulter, Brea, CA, USA). The concentration and quality of the prepared libraries were assessed using a Qubit 4 fluorimeter (Thermo Fisher Scientific, Waltham, MA, USA) and a Nanofor 05 automatic genetic analyzer (Syntol, Moscow, Russia). After combining the libraries at equimolar ratios into 16S rRNA and ITS pools, their quality was assessed on an Agilent 2200 TapeStation system (Agilent Technologies, Santa Clara, CA, USA). Sequencing of the library pools was performed on an Illumina NovaSeq 6000 system (2 × 250 paired-end reads) (Illumina, San Diego, CA, USA). Demultiplexing and conversion to FASTQ files were performed using the BCL Convert v4.2.4 software (Illumina). The raw sequences were deposited into GenBank under accession number PRJNA1365504.

### 4.3. Bioinformatics Analysis

Amplicon sequencing data analysis was performed in QIIME 2 Framework using Amplicon v. 2025.4 suite of plugins [56]. Error correction, paired-end read merging, and assignment of amplicon sequence variants (ASVs) were performed using the dada2 plugin [57]. ASVs found in only one sample were removed. For taxonomic annotation, we used the q2-feature-classifier plugin and classify-sklearn method [58]. The taxonomic classifier for identifying bacteria was trained on diverse weighted Silva v. 138 99% OTUs full-length sequences [59,60,61,62,63], and the classifier for identifying fungi was trained on UNITE v. 10 2025-02-19 dynamic all eukaryotes including singletons [64,65]. Metazoa, Protista, Viridiplantae, and unassigned (up to the division level) sequences were filtered out of the ITS sequencing results. Chloroplast, mitochondria, Eukaryota, Archaea, *Asinibacterium* (synonym *Sediminibacterium*, contaminant [66]) and unassigned (up to the phylum level) sequences were filtered out of 16S rRNA sequencing results. Fungal and bacterial ASVs were collapsed at the genus level. After taxonomic filtering, samples with fewer than 20 ASVs were removed from data sets.

Alpha diversity for groups of samples with and without decline symptoms were calculated using the Shannon index (q2-diversity plugin). Each sample selection was composed of samples of the same variety, location, and year of sampling. To assess differences between samples, we used the pairwise Kruskal–Wallis test. Differential abundance testing was performed using the ANCOM-BC (q2-composition plugin). The division of fungal genera into ecological groups was performed using FungalTraits database [67] and their role in grapevine diseases. To construct histograms of the distribution of ecological groups, each fungal genus was counted for both primary and secondary lifestyles. If only a primary lifestyle was specified in the database, it was counted twice. Data visualization was performed using the q2-vizard plugin and the ggplot2 v. 4.0.0 R package.

Beta diversity analysis was performed using MicrobiomeAnalyst [68]. Differences in microbial community composition between grapevines with and without decline symptoms were assessed using Principal Coordinates Analysis (PCoA) based on the Jaccard dissimilarity index. The raw data for the analysis was the relative abundances of bacterial and fungal taxa. Genera with an abundance below 0.1% were excluded from these analyses. Statistical significance of group differences was evaluated using PERMANOVA.

### 4.4. Detection of Phytoplasmas

For detection of phytoplasmas, real-time PCR was used. Reactions were performed in triplicate using a RealTime DTprime amplifier (DNA-Technology, Moscow, Russia) and the BioMaster HS-qPCR reagent kit (Biolabmix, Novosibirsk, Russia) according to the manufacturers’ recommendations. The same DNA samples used for preparation of libraries for amplicon sequencing were used for the PCR analysis. DNA concentration was measured using a Qubit 4.0 fluorimeter (Invitrogen, Waltham, MA, USA) and then diluted to a working concentration of 5 ng/µL. The amount of DNA in the reaction mixture was 20 ng. PCR was performed using both universal primers (UniRNA) targeting the 16SrRNA gene region of the Candidatus Phytoplasma genus [69] and primers specific to the 16SrV group phytoplasma, including Grapevine flavescence dorée phytoplasma (FD) according to the TaqMan^®^ multiplex PCR protocol described by Angelini et al. [70]. As an endogenous control, the grapevine chloroplast chaperonin 21 gene was used. A PCR result was considered positive if the amplification reaction was successful in two or three replicates.

## 5. Conclusions

Studying the complex of pathogens associated with grapevine decline is essential for solving two problems: establishing the etiology of the disease and developing effective vineyard protection systems. The obtained metagenomic data on the taxonomic composition and structure of the grapevine microbiome confirm the presence of a complex of suspected pathogens on affected vines. These findings open up opportunities for developing preventative protection strategies, shifting the focus from managing the consequences to early warning of threats, which is critical for the sustainable development of viticulture in the future.

## Data Availability

The raw sequencing data are available in the NCBI SRA repository under the BioProject accession number: PRJNA1365504.

## Supplementary Materials

The following supporting information can be downloaded at: https://www.mdpi.com/article/10.3390/plants14243722/s1, Figure S1: Sampling locations; Figure S2: Functional properties of the fungal communities in grapevine with and without decline symptoms; Figure S3: Bacterial community composition in Avgustin grapevines at genus level with relative abundance > 1%; Figure S4: Bacterial community composition in Cristal grapevines at genus level with relative abundance > 1%; Figure S5: Bacterial community composition in Riesling grapevines at genus level with relative abundance > 1%; Figure S6: Principal Coordinates Analysis (PCoA) of microbial community structure in grapevines with and without decline symptoms; Table S1: Grapevine samples and symptoms description; Table S2: Number of reads and ASVs obtained after amplicon sequencing; Table S3: Results of taxonomic classification of bacterial ASVs after filtering; Table S4: Results of taxonomic classification of fungal ASVs after filtering; Table S5: Functional properties of the fungi; Table S6: The result of real-time PCR detection of phytoplasmas in samples.
