# Supplementary material for: Metagenomic Study of the Grapevine Decline Detected a Cocktail of Fungi Associated with Grapevine Trunk Diseases
Source: Plants (Basel). 2025 Dec 5;14(24):3722. doi: 10.3390/plants14243722 (PMC12737265; doi:10.3390/plants14243722)
Supplement: Supplementary file 1 [file plants-14-03722-s001.zip › Suppl_Figures.pdf]

Supplementary materials, Karpova et al., 2025

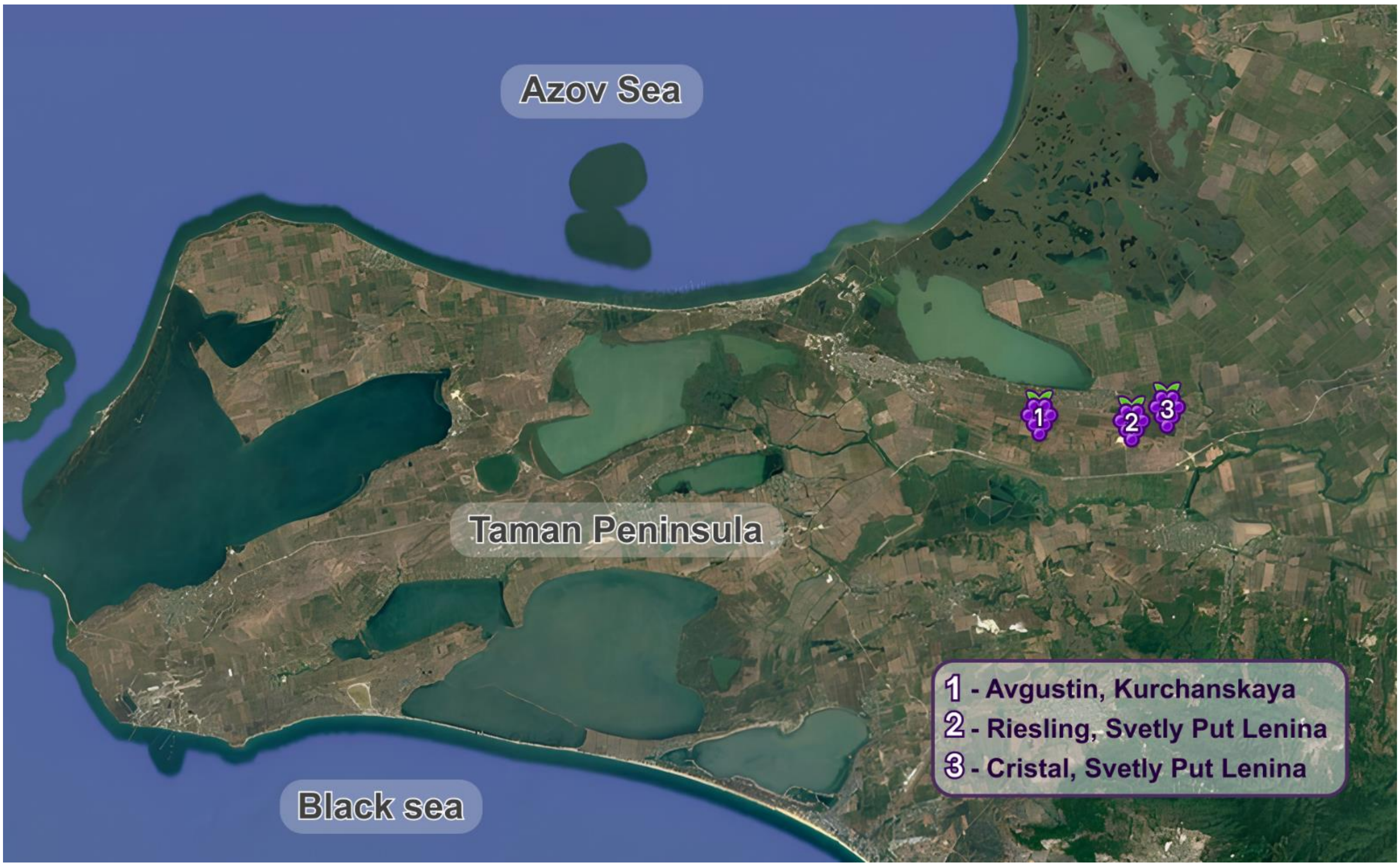

Figure S1. Sampling locations.

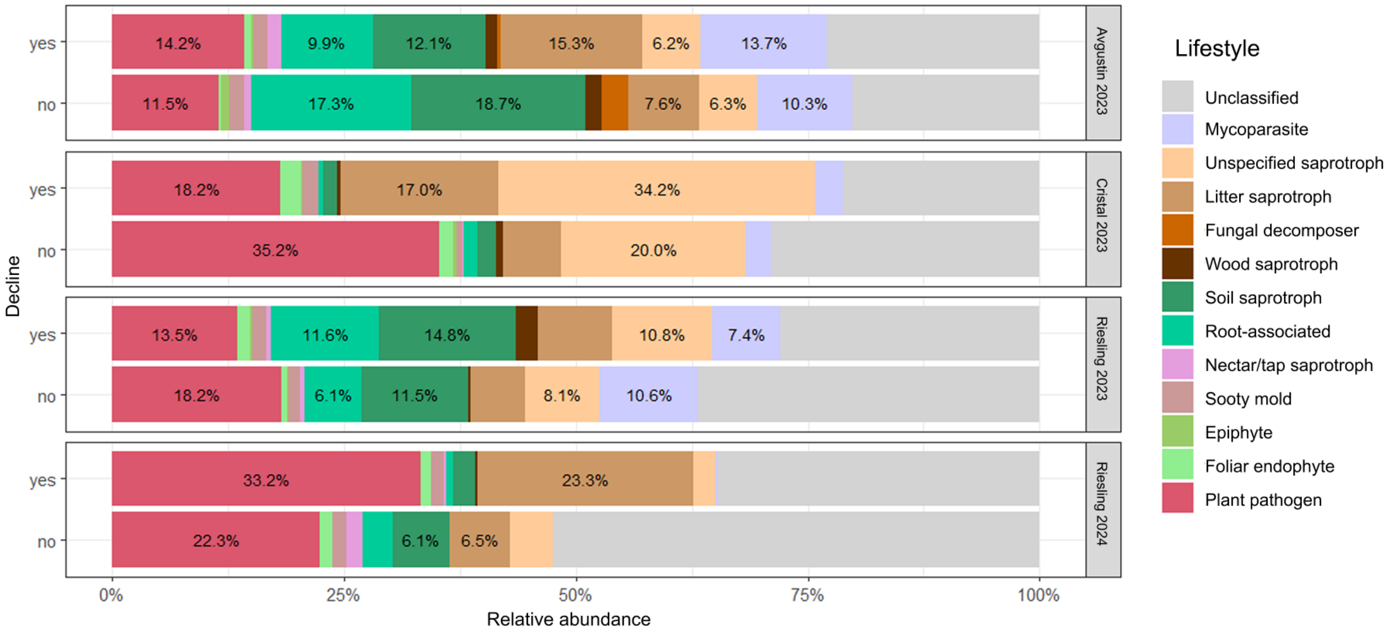

Figure S2. Functional properties of the fungal communities in grapevine with and without decline symptoms. The diagram is based on the relative abundance of each fungal genus in each sample. The FungalTraits database was used to assign fungi to groups according to their lifestyle (Table S5).

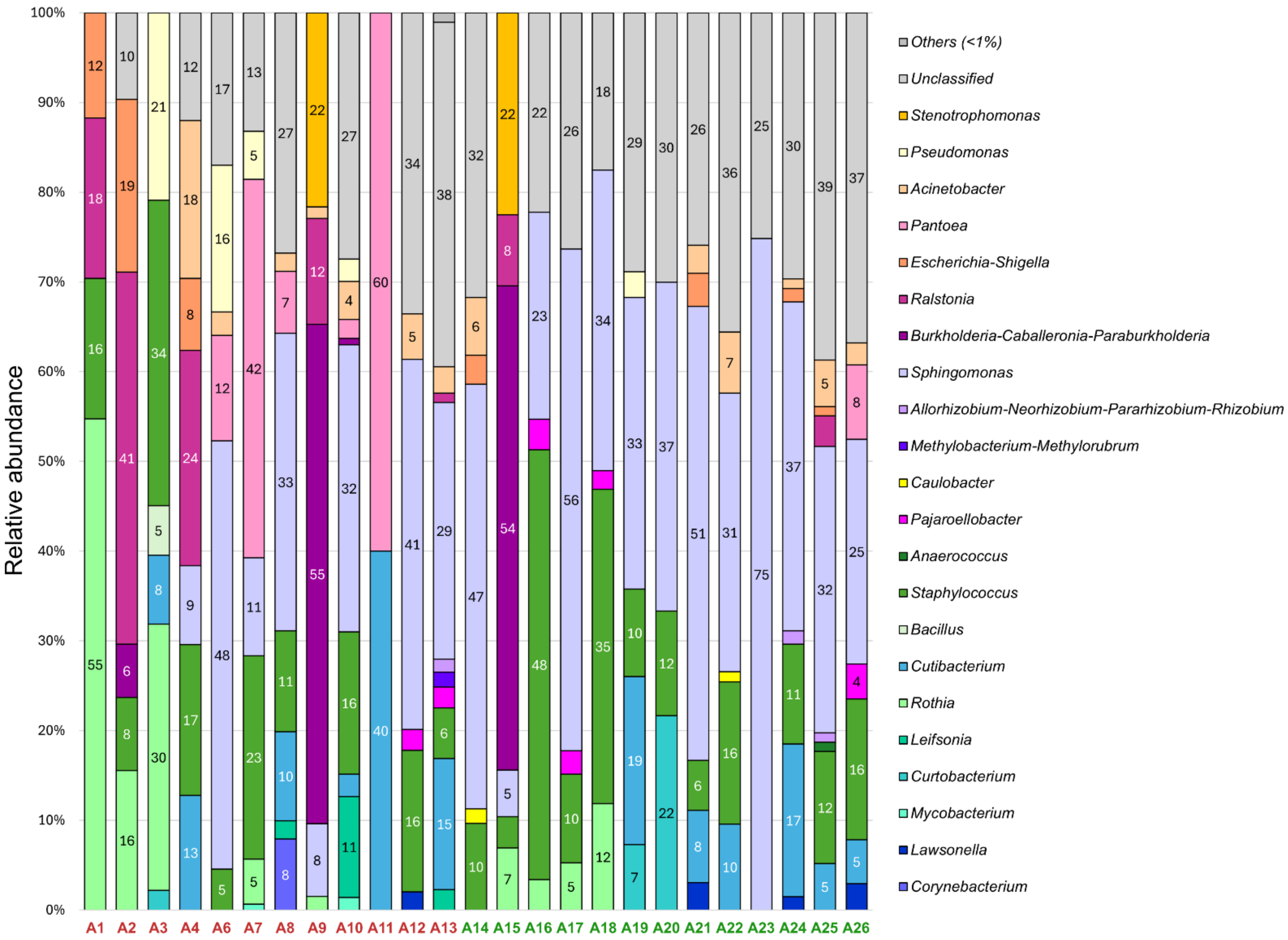

Figure S3. Bacterial community composition in Avgustin grapevines at genus level with relative abundance > 1%. Samples with (A1-A13) and without (A14-A26) decline symptoms are marked in red and green, respectively.

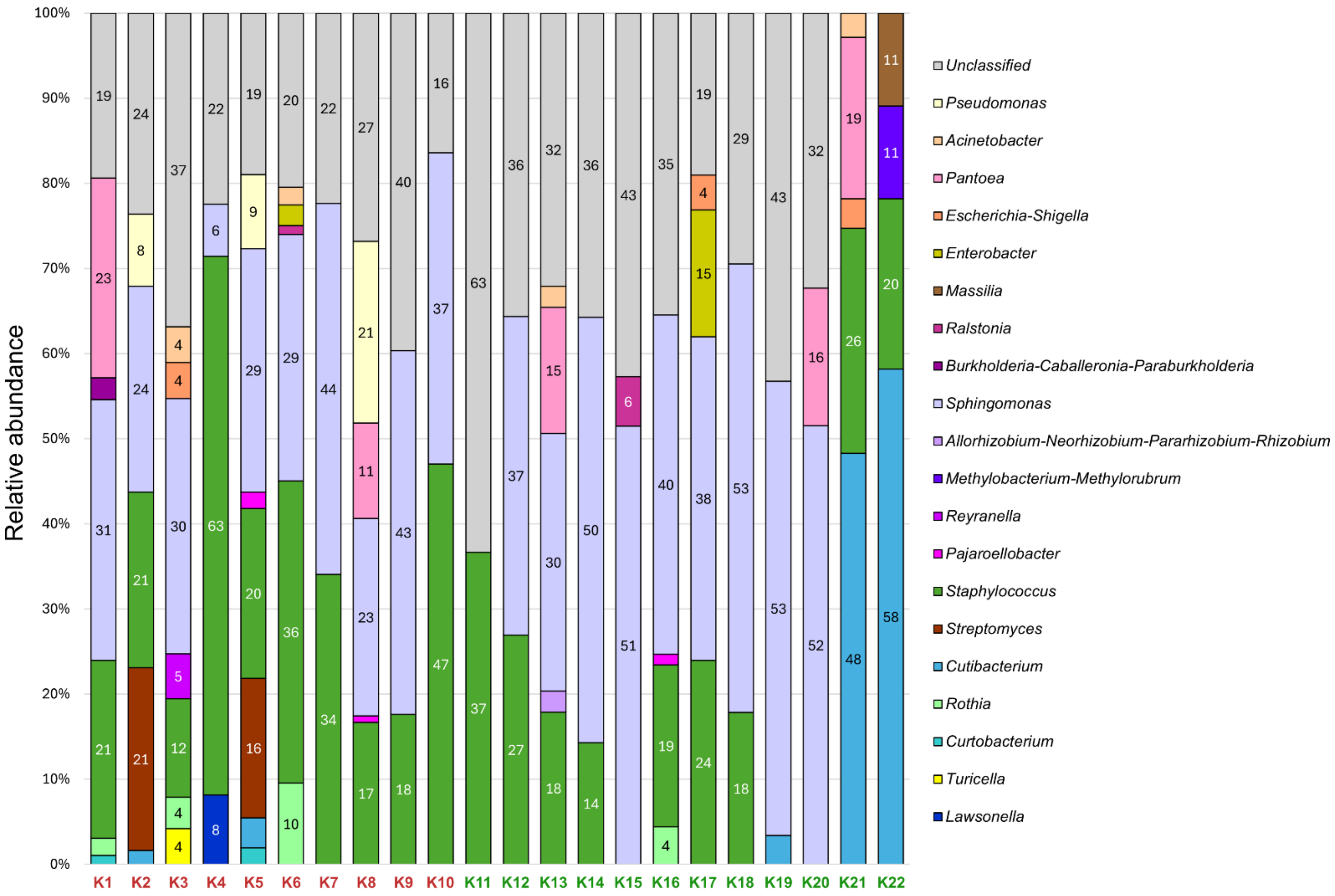

Figure S4. Bacterial community composition in Cristal grapevines at genus level with relative abundance > 1%. Samples with (K1-K10) and without (K11-K22) decline symptoms are marked in red and green, respectively.

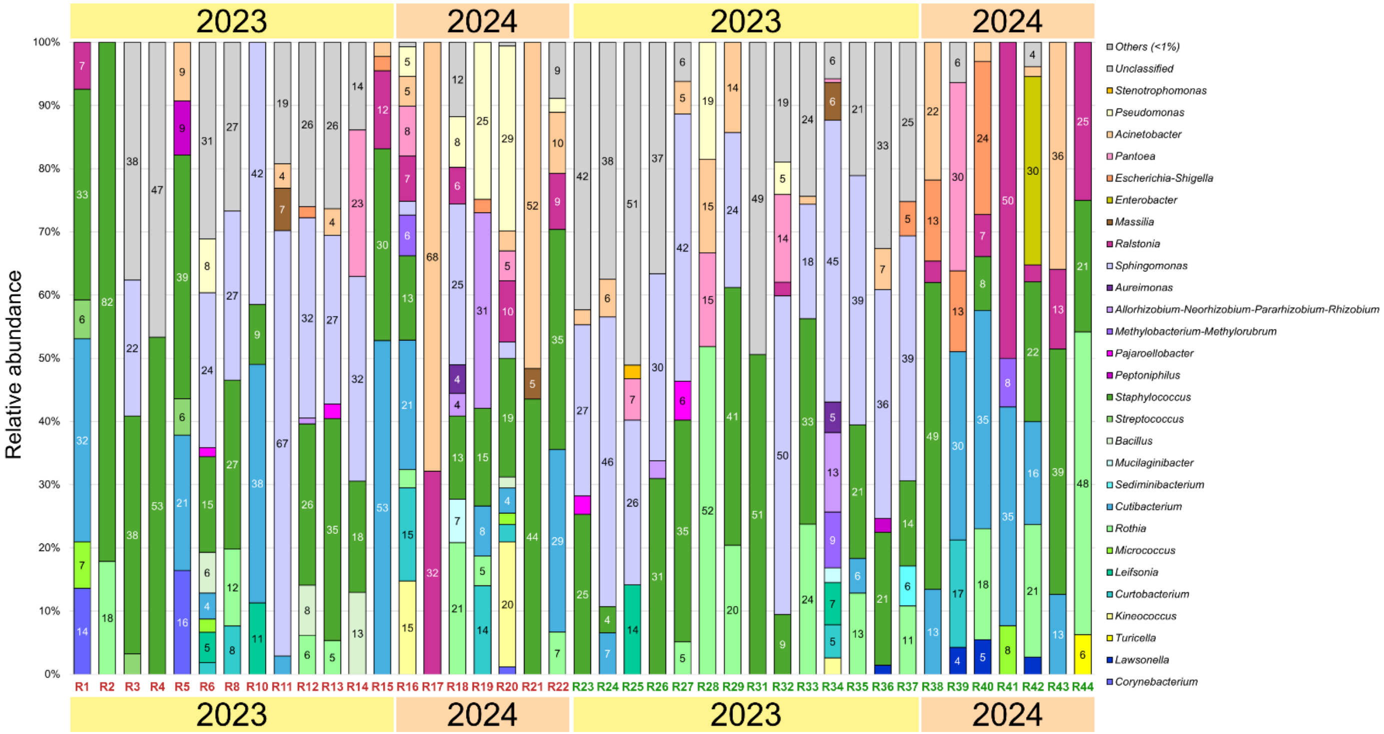

Figure S5. Bacterial community composition in Riesling grapevines at genus level with relative abundance > 1%. Samples with (R1-R22) and without (R23-R44) decline symptoms are marked in red and green, respectively.

PERMANOVA (with decline vs without decline)

	Avgustin 2023	Cristal 2023	Riesling 2023	Riesling 2024
Num. of samples	13 vs 13	10 vs 10	15 vs 15	7 vs 7
F-value	1.5568	1.9521	1.1565	1.2528
R^2	6.1%	9.8%	4.0%	9.5%
p-value	0.034	0.006	0.18	0.077

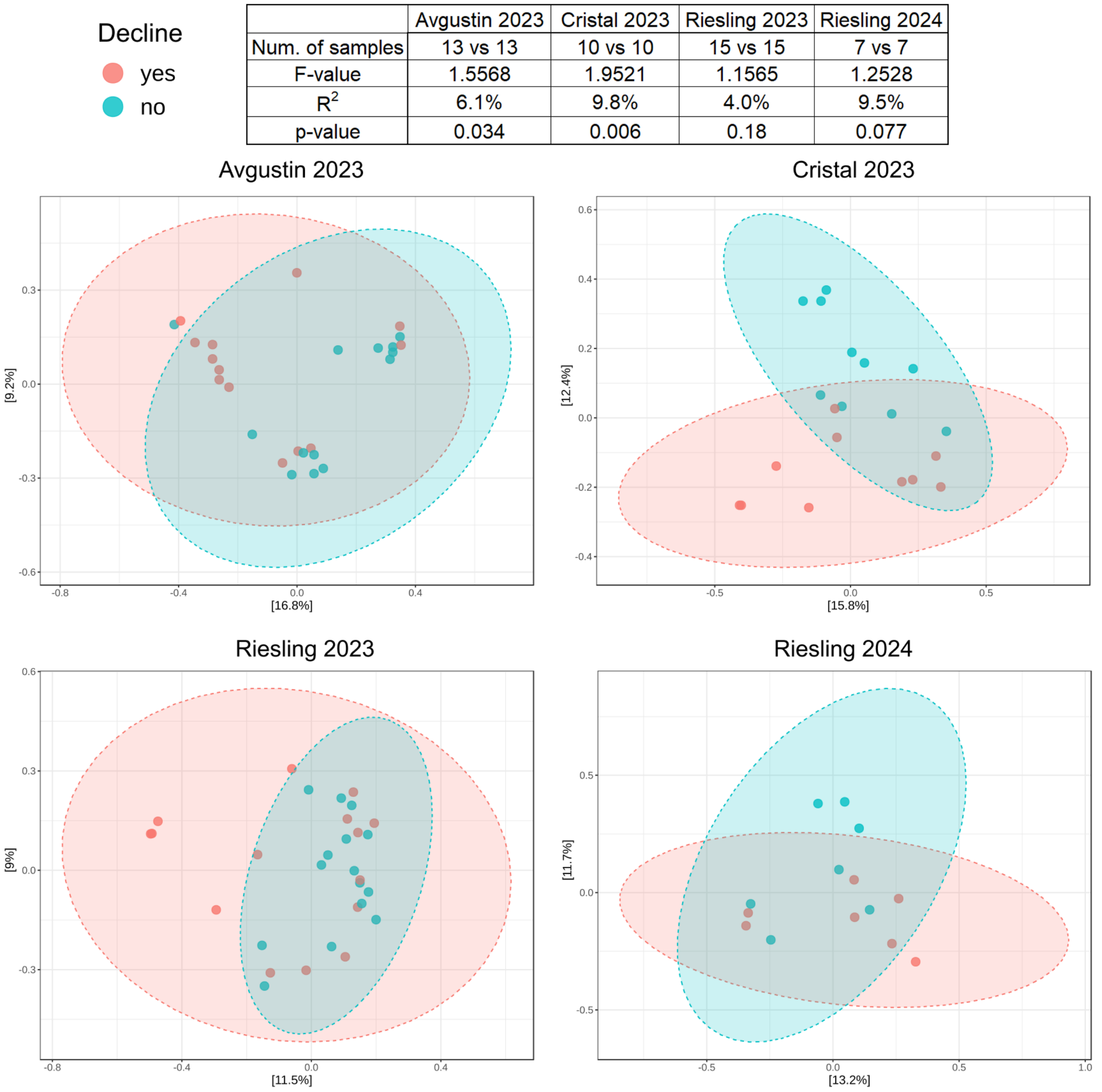

Figure S6. Principal Coordinates Analysis (PCoA) of microbial community structure in grapevines with and without decline symptoms. PCoA plots based on the Jaccard distance. PERMANOVA results (top table) show differences in the structure of microbial communities in samples with and without decline symptoms.
